# Mixing patterns and the spread of close-contact infectious diseases

**DOI:** 10.1186/1742-7622-3-10

**Published:** 2006-08-14

**Authors:** WJ Edmunds, G Kafatos, J Wallinga, JR Mossong

**Affiliations:** 1 Statistics, Modelling and Bioinformatics Department, Health Protection Agency Centre for Infections, 61 Colindale Avenue, London NW9 5EQ, UK; 2 Department of Infectious Diseases Epidemiology, National Institute of Public Health and the Environment (RIVM), PO Box 1, 3720 BA Bilthoven, The Netherlands; 3 Laboratoire National de Santé, P.O. Box 1102, L-1011, Luxembourg

## Abstract

Surprisingly little is known regarding the human mixing patterns relevant to the spread of close-contact infections, such as measles, influenza and meningococcal disease. This study aims to estimate the number of partnerships that individuals make, their stability and the degree to which mixing is assortative with respect to age. We defined four levels of putative at-risk events from casual (physical contact without conversation) to intimate (contact of a sexual nature), and asked university student volunteers to record details on those they contacted at these levels on three separate days. We found that intimate contacts are stable over short time periods whereas there was no evidence of repeat casual contacts with the same individuals. The contacts were increasingly assortative as intimacy increased. Such information will aid the development and parameterisation of models of close contact diseases, and may have direct use in outbreak investigations.

## Background

Mathematical models have long been used to further our understanding of the spread of close-contact infections, such as measles and tuberculosis, and make quantitative predictions regarding the possible impact of control policies [1-4]. Largely through the development and exploration of these models the pivotal role of the patterns of host mixing to the spread of such pathogens has been acknowledged [2,4-7]. Broadly, the models employed by epidemiologists can be subdivided into two [8], according to their assumptions regarding the stability of contacts: those that employ the mass-action assumption, or variants of this [1-6], in which contacts are instantaneous and independent i.e. once contacted, individuals are no more (or less) likely to contact each other in the future; or network models and their variants in which individuals are thought of as the nodes in a network and the links between them their contacts [9-11]. These models tend to assume that individuals form relatively stable partnerships (some, indeed, assume a static network). Micro-simulation, or agent-based models (e.g. [10]) in which individuals are often assumed to reside in households travel to schools or workplaces daily and so on, fall into the latter category of models in which individuals are (often implicitly) assumed to form relatively stable contact networks.

Which of these classes of model best fits reality: one in which at least some of the contacts are stable (such as network models), or one in which contacts are transient and constantly changing (as in mass action variants)? Does this alter for different close-contact infections? Furthermore, are the characteristics of those that are contacted random or is there a degree of preferred, or assortative mixing?

Models of close-contact infectious disease (of all types) tend not to be parameterised by directly analysing mixing pattern data. Instead, assumptions are made about mixing and the models are then calibrated to epidemiological data. These assumptions might be very simple (e.g. homogenous mixing) or they may be very detailed, such as with most micro-simulation models. In both cases the epidemiologically relevant mixing patterns are assumed and then estimated. However, there is a lack of data on which to base these assumptions, and the number of mixing parameters to be estimated often exceeds the number of epidemiological data points against which the model is calibrated (i.e. there is often an identifiability problem). Since the patterns of mixing are crucial to determining the spread of these infections (they can only be spread via contact), the lack of an empirical base on which the models are based is problematic.

This study aims to explore these issues of both appropriate model structure and parameterisation by asking individuals to record details of those that they contacted in diaries [12]. The results should allow a deeper understanding of the routes of transmission, improve the parameterisation of models, and aid the investigation of outbreaks of close-contact pathogens.

## Analysis

### Methods

A convenience sample of undergraduate students at the University of Warwick were asked to record details of the persons they had contact with for three days. The contacts were recoded during a two-week period during the spring term. As individuals tend to make similar numbers of contacts during weekdays, but fewer during the weekend [12], individuals were over-sampled during the weekend. Hence, participants were randomly assigned one weekday and both weekend days or alternatively, two randomly assigned weekdays and one randomly assigned weekend day. The participants were told in advance which days they had been assigned (rather than asked to remember their contacts some days ago) and were encouraged to fill out the form during the day. The forms were collected after the third day. In practice, the final day of the survey was always a weekend when individuals would be expected to make fewer contacts than during the week. Thus, the cumulative number of contacts made would be expected to saturate as a result of the final sampling occasion always being a weekend. To avoid biasing the results regarding the acquisition of new contacts over time, in the analysis the order in which the participants filled out the survey was randomised so that the third day on which the survey was filled out (a weekend) may have been analysed as if it had been the first or second day.

The questionnaire recorded details of participants' age, sex, and living and working arrangements. They were then asked to record the age and sex along with a unique identifying code for each of the persons they contacted during each study day. It was stressed to the participants that they should maintain the same code for each person (in practice the initials of the contacts were almost exclusively used). They were asked to record in which social context the contact took place and the intimacy level of that contact. Four different intimacy levels were defined: physical contact without conversation (Level 1); conversation without physical contact (Level 2); conversation with non-sexual physical contact (Level 3); and any sexual contact including kissing of a sexual nature (Level 4). These levels of intimacy were chosen as they were simple to remember and record and easily distinguishable from each other; and they were designed to reflect the different kinds of behavioural patterns important for the spread of a range of close-contact infections. If the individual had contact with the same person more than once in a given day they were asked to record the contact only once in the context and place in which they had the most intimate contact. Note that the survey was of individuals contacted not of every contact. This is analogous to recording partnerships rather than sex acts for estimating the characteristics of sexual contact networks. A conversation for the purposes of the study was defined as a situation in which either party said a single word or more, at a distance that did not require voices to be raised and in the absence of physical barriers such as security screens. The length of each contact was not recorded nor was the number of contacts made with the same person during that day. A day was defined as the time period between getting up and going to bed (rather than 24 hours). Individuals were encouraged to report any difficulties they experienced in filling out the questionnaire.

Given that contacts are made by individuals on different days multilevel modelling was used to take account of the hierarchical structure of the data, and the non-independence of observations (assuming independence of observations would lead to underestimation of standard errors). The statistical analysis was divided into two parts investigating (a) the number of contacts per day per individual and (b) the age difference between participants and contacts. For the first part of the analysis a two level regression model was fitted using sampling day as level 1 and participants as level 2. The analysis of the difference between age of individuals and those contacted was carried out using a 3-level model with contact as level 1, weekday/weekend as level 2 and individual as level 3. The absolute difference between the age of individual and the age of those contacted was taken as the response variable. A model was initially fitted with contact intensity (physical, conversation, conversation and physical and sexual), sex (same sex, different sex between the individual and the person contacted) and day of the week (weekday, weekend). The least significant factor was dropped from the model using the likelihood ratio test in a stepwise fashion until all the remaining variables were significant (p < 0.05). The same process was repeated with place (home, college, travel, shop, social and other) instead of contact intensity because of the colinearity between the two variables. The multilevel analysis was carried out using gllamm in Stata Statistical Software (Release 8.2 College Station Texas, USA. StataCorp, 2003).

The Health Protection Agency investigates and records outbreaks of selected infectious diseases in England and Wales. The difference in age between primary and secondary cases (defined by date of onset) was compared for clusters of measles and meningococcal disease [13] occurring over the period 1995–1998 (just prior to the introduction of meningococcal C vaccination in the UK). Measles outbreaks in Steiner communities were excluded, as it is not uncommon within these communities to deliberately infect susceptible children [14].

## Results

Of the 35 individuals asked to participate in the survey, 29 (83%) completed the questionnaire, 15 females and 14 males. One male was excluded from the analysis as he was not a full-time student (hereafter all results exclude this person). The ages of participants ranged from 18 to 24 years with a mean age of 20.5 years. Six first year undergraduates (aged 18–19 years) and 22 third year undergraduates (aged 20–24) participated in the survey.

The 28 individuals recorded a total of 1792 contacts. Of these, 9 were dropped from subsequent analysis since they were reported twice on the same day. A summary of the contacts at the different levels of intensity is given in table 1 (a and b). On average individuals made 26 contacts per day during the week and 19 per day during the weekend, a difference that was statistically significant (Table 2). There were no significant differences in the number of individuals contacted by sex or living arrangements (Table 2). Interaction terms were also tested, but none were significant. Seventy-five percent (1340) of the people contacted were only contacted on one of the study days.

**Table 1:** a-b. Summary tables of contacts by intensity of contact. a) the average number of contacts made by day of week and b) social context of contacts.

a)								
	**Mon**	**Tue**	**Wed**	**Thu**	**Fri**	**Sat**	**Sun**	***Total***
**Level 1**	1.3	0.5	2.0	1.0	0.4	0.7	0.2	*61*
**Level 2**	14.9	12.5	12.6	18.0	14.8	13.1	8.1	*1085*
**Level 3**	7.6	11.9	8.0	7.3	9.1	6.6	3.6	*605*
**Level 4**	0.6	0.5	0.6	0.3	0.3	0.4	0.3	*32*
***Total***	*24.4*	*25.4*	*23.2*	*26.5*	*24.7*	*20.9*	*12.2*	*1783*
								
b)								
	**Type of contact**							

**Context**	Level 1		Level 2		Level 3		Level 4	

Home (n = 690)	3%		32%		51%		84%	
College & Social (n = 704)	44%		41%		38%		16%	
Travel & Shop (n = 272)	51%		19%		5%		0%	
Other (n = 126)	2%		8%		6%		0%	

**Table 2:** Multivariable analysis of daily number of contacts

**variable**	**level**	**coefficient**	**95% Confidence Interval**	**p-value**
constant		26.05	(16.76, 35.34)	<0.001
sex	male	baseline		
	female	-1.44	(-5.30, 2.42)	0.466
accommodation	Student halls	baseline		
	other	-3.90	(-10.14, 2.34)	0.228
# of people in household*		0.32	(-0.55, 1.20)	0.470
weekday/weekend	weekday	baseline		
	weekend	-6.87	(-10.36, -3.38)	<0.001

* Defined as "sharing a kitchen"

Note: individual and day of week, were random effects, sex, accommodation, # people in household, and weekday/weekend were modelled as fixed effects. Degrees of freedom = 1748.

**Figure 1:**
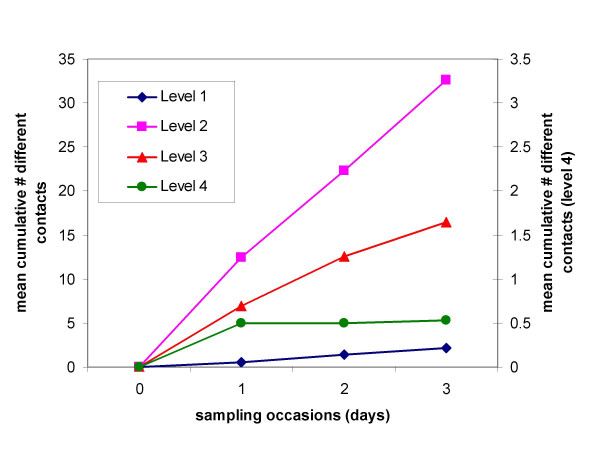
The mean cumulative number of *different *individuals contacted over the three study days (sampling occasions) by contact intensity. Note that as the number of Level 4 contacts was smaller than the other types of contact they are plotted on a different scale (right-hand axis).

Figure 1 shows the mean cumulative number of different individuals contacted over the three days at each of the intensities (note that the results for Level 4 are plotted on a different scale). It is clear that there are very different patterns in the acquisition of new "partners" for the different levels of contact. The students in this study recorded approximately 0.7 Level 1 contacts (physical contact without conversation) per day and these contacts were with different individuals from day to day (as indicated by the linear increase in the cumulative number of contacts). That is, there was no evidence that, for this level of contact, they started to have repeat contacts with the same individuals over the study period. In contrast, half of the participants reported a Level 4 contact on the first sampling occasion but there was only one new individual contacted (at this level) during the remaining sampling occasions (note that each Level 4 contact was contacted twice, on average, over the three days). The pattern in the acquisition of new "partners" for the other two levels of contact were intermediate between the two extremes.

The data presented in Figure 1 were extrapolated to estimate the average number of different contacts that would be expected over the period of a week (typical infectious period for diseases such as measles and chickenpox). This revealed that on average 5 different individuals would be contacted at Level 1 over this time period, 60 at Level 2, 17 at Level 3 and 0.5 at Level 4. Thus, if an infection can be transmitted via conversational contact then the number of effective contacts that an individual from this population would make over the period of a week would be approximately 78 (60 + 17 + 0.5).

**Figure 2:**
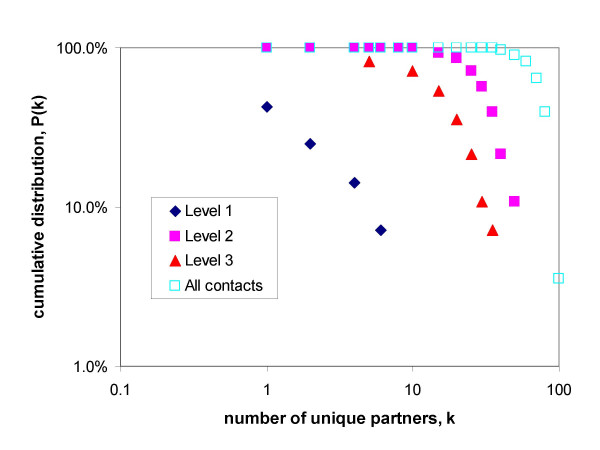
Log_10_-log_10 _plot of the cumulative distribution of the total number of unique contacts made at Levels 1–3 and all contacts over the three sampling days.

Figure 2 is a Log10-log10 plot of the cumulative distribution of the total number of unique contacts made at Levels 1–3 over the three sampling days. Level 4 was excluded as individuals either made 0 or 1 contacts at this level. A linear relationship on a log-log scale would suggest that there is a "fat tail" to the distribution – that is a small proportion of the people make a large proportion of the contacts. There is little evidence to suggest that this is true for any of the levels of contact, though the data on Level one (Physical contact only) is very sparse, and the population sampled (university students) was relatively homogenous.

In this university setting mixing is highly assortative (like-with like) with respect to age. The mean age of those contacted was 22.3, 25.0 and 29.7 years for 18–19 year olds, 20–21 year olds, and 22–24 year olds respectively. Outside their own age group, participants contacted individuals in older age groups, but had very little contact with those in the age groups below them. For instance, only 10 of the contacts were with individuals less than 15 years of age, whereas 273 were with individuals 30+ years of age.

**Figure 3:**
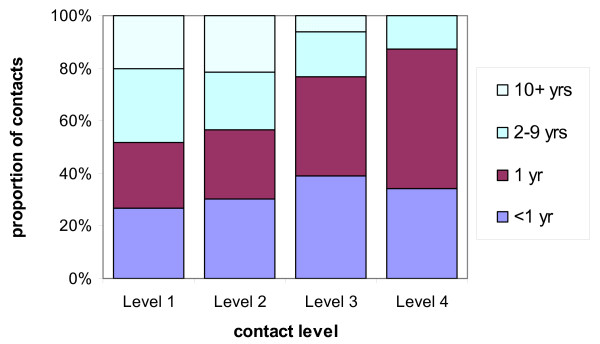
The difference in age between participants and their contacts by intensity of contact.

Figure 3 suggests that the degree of assortativeness of mixing (with respect to age) increases for the more intimate contacts. For instance, 85% of contacts at Level 3 and 4 were with individuals within 2 years of age of the study participant, whereas only 6% of Level 3 contacts and no Level 4 contacts were made between individuals 10 years or more different in age. In contrast, only 62% of contacts at Level 1 and 2 were within 2 years of age of the study participant. The intensity of contact was the only factor that remained significant in the model (Table 3a). The pattern of increasing age clustering of contacts with increasing intimacy was observed in almost all individuals. Correlation coefficients between the absolute value of the age-difference, and the level of contact (1,2,3 or 4) were negative for 24 out of the 28 participants. That is, the higher levels of contact were associated with a smaller age difference.

**Table 3:** Factors associated with age difference between the individuals and those that were contacted, results of multivariate analysis of most parsimonious model.

a)				
**variable**	**level**	**coefficient**	**95% Confidence interval**	**p-value**

constant		10.20	(7.56, 12.84)	<0.001

intensity	Physical only	baseline		
	Conversation only	-1.10	(-3.64, 1.44)	
	Conversation & physical	-4.73	(-7.32, -2.13)	
	"Sexual" contact	-7.75	(-11.92, -3.58)	<0.001
				
b)				

**variable**	**Level**	**coefficient**	**95% Confidence interval**	**p-value**

constant		1.58	(0.60, 2.55)	0.002

Place of contact	Home	baseline		
	College	5.28	(3.92, 6.64)	
	Travel	7.30	(5.22, 9.38)	
	Shop	13.81	(12.24, 15.38)	
	Social	2.34	(1.09, 3.59)	
	Other	5.19	(3.36, 7.03)	<0.001

Note: contact, individual and day of week, were random effects, intensity and place of contact were modelled as fixed effects. Degrees of freedom = 1783 and 1776 for Table 2a and b respectively.

Not only were different levels of contact associated with different degrees of assortativeness but also the different types of contact occurred in different social environments. As expected, the more intimate the contact, the more likely it was to occur at home, or in a "social" context (Table 1b). Conversely Level 1 contacts were most likely to occur while shopping or travelling. The model was therefore re-fitted replacing the intensity of the contact with the place the contact took place. On average there were 1.5 years difference when the contact occurred at home, 4 years when socialising, 7 years when the contact occurred at college, 9 years during travelling, 15.5 years when shopping, and 7 years in other places (Table 3b).

**Figure 4:**
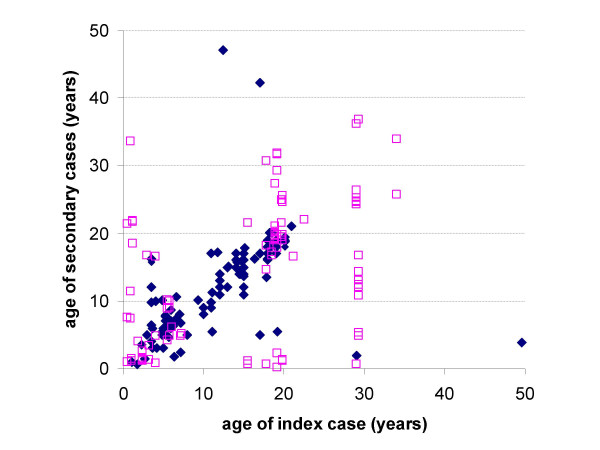
The age of primary and secondary (based on date of onset) cases of measles (open squares) and meningococcal meningitis (filled diamonds) in clusters in England and Wales 1995–1998.

To assess whether this pattern of increasing assortativeness with increasing degree of intimacy is reflected in different patterns of infection we compared cluster data on the age of primary and secondary cases of measles and meningococcal disease in England and Wales. The data suggest a closer age-clustering of secondary cases for meningococcal disease than for measles (Figure 4). The mean absolute difference in age between primary and secondary cases of meningococcal disease was 3 years (Standard Deviation = 6.5, n = 123), which compares with a mean absolute difference of 7.7 years (SD = 8.3, n = 113) for measles.

## Discussion

The early models of human immunodeficiency virus transmission tended to be very inaccurate, partly because of a lack of data on sexual behaviour. This stimulated a large number of detailed surveys of patterns of sexual mixing in many different countries (e.g. [15-17]) that has lead to a deeper understanding of the spread of sexually-transmitted infections and improved model-based predictions. No comparable research effort has been initiated for close-contact infections, even though many of the major health threats that face us today (such as pandemic influenza or severe acute respiratory syndrome), and the endemic diseases that cause such morbidity worldwide (such as pneumococcal disease, influenza and measles) are spread via this route. This diary-based study is a small attempt to try and fill this void. It shows that it is possible to quantify the stability and assortativeness of mixing patterns for differing types of contact. This may aid the construction and parameterisation of realistic mathematical models of the transmission of close-contact infectious diseases and the analysis of outbreaks. The use of a convenience sample has helped us to investigate the patterns of contact, but clearly limits the generalisability of the results to the population at large.

There are a number of limitations inherent to this study, which should be borne in mind. First, the exact nature of at-risk events is largely unknown. The contacts, as defined, probably do not represent actual at risk events, though they may share characteristics with them. Thus, for instance, the definition of Level 1 probably fails to pick up all the relevant contacts at this level for infections such as measles (e.g. individuals on a crowded bus), but we may speculate that these (unknown) contacts might have similar characteristics (age, sex, the likelihood of contacting them again) to those who actually were contacted. That is, those that were contacted are a (random) sample of those available to contact at this level. In addition, although recorded contacts are presumably only proxies of real at-risk events, a recent paper has suggested that they offer a better explanation of observed incidence of mumps and influenza than common mass-action assumptions [18]. In addition, the definitions used take no account of repeat contacts with the same individual in a day, or the length of time over which the contact took place. In essence we are enumerating the different partnerships not the number of at-risk events per partnership.

Second, although the survey was well accepted, the quality of the resulting data is difficult to judge, as there are so few similar studies. The number of individuals contacted per day is similar to that reported in our previous study [12] (17 contacts) and by de Sola Pool and Kochen [19] (23 contacts per day including telephone conversations and letters as well as face to face communication), as well as a recent survey of (primarily) university students in Belgium [20] (18 contacts during weekdays, 12 at the weekend). Furthermore, the acquisition of new contacts over time reported by de Sola Pool and Kochen was similar in shape to that shown in Figure 1 (i.e. increasing rapidly then gradually saturating). Thus the dynamic patterns of contacts and the number of contacts that individuals make reported here appears to be broadly consistent with the few other studies available in the literature.

The accuracy of diary-based reporting of contacts has been questioned [21,22], and a few students reported difficulties in remembering all their contacts, particularly those at Level 1 and Level 2 in the social context. Indeed, comparison of self-reported diaries of telephone conversations with data from a digital exchange found that shorter conversations were less likely to be recorded [21]. Thus, it seems that under-recording is more likely to have occurred at the lower intensity of contact. From our experience accuracy of reporting is greatly improved by ensuring that individuals know in advance when they are to report their contacts, rather than relying on them to remember them afterwards, although this was not the case in the Belgian study [20]. Other methods of estimating contact patterns have been proposed, such as using travel-to-work data (e.g. [10]) or mobile telephones or other devices to track individuals. These techniques do not, however, provide information on contacts and their intimacy. They merely provide data on the location of individuals. Diaries have the potential to provide much more detailed and relevant contact information. There is, however, a clear need to test the validity of this approach by linking self-reported mixing patterns to evidence of exposure to infection.

One of the most striking findings of this work was that mixing patterns were more like-with-like with respect to age at the higher intensity levels. This has important implications for the construction and parameterisation of models of disease transmission and for outbreak control. With regards to model construction, the results suggest that mixing patterns should be more assortative for infections which require more intimate contact to effect transmission. With regards outbreak control, it seems apparent that secondary cases are more likely to be closer in age to a primary case for infections that require more intimate contact. Analysis of the meningococcal and measles clusters data lends support to this hypothesis.

The pattern of acquisition of new partners (Figure 1) provides an insight into the structure and dynamics of relevant contact networks. Clearly these networks are not static since the total number of individuals contacted increased with the number of sampling opportunities for all levels of contact except the most intimate (which would also be expected to increase over a longer time-scale). It is interesting that the four levels of contact we defined display a range of patterns, such that there was no evidence to suggest that the rate of acquisition of Level 1 contacts slowed over the period of study, whereas the number of Level 4 contacts saturated almost immediately. The other two levels of contact showed intermediate patterns. Thus it would seem that the more intimate the necessary contact for transmission, the more appropriate it would be to model using a neighbourhood of relatively stable contacts. That is, network models may be more appropriate for infections that require intimate contact, whereas mass action models (or their variants) may be appropriate for those diseases that can be spread via casual contact.

## Conclusion

We have demonstrated that it is possible to obtain relevant information on contact patterns for close-contact infections by the use of self-reported diaries. The contact patterns that emerge appear to depend on the degree of intimacy required for transmission to occur. Such information can help guide which models are most appropriate for investigating the behaviour of different infectious diseases, aid the parameterisation of these models, and be of direct use in outbreak investigations.

## Competing interests

The author(s) declare that they have no competing interests.

## Authors' contributions

WJE conceived, designed and directed the study, and took the lead in writing the manuscript. GK performed the statistical analysis of the data, and contributed to writing the mansucript. JM and JW contributed to the design of the analysis of the data, read and critically contributed to the write-up.
